# Canine and Feline Melanoma: Diagnosis, Treatment, and Prognosis

**DOI:** 10.3390/vetsci13101053

**Published:** 2026-10-08

**Authors:** Haein Jeong, Minyeong Shin, Yujin Kim, Minseo Ko, Dong-In Jung, Kyu-Woan Cho, Hyeona Bae

**Affiliations:** College of Veterinary Medicine, Gyeongsang National University, Jinju 52828, Republic of Korea; jhaein77@gmail.com (H.J.); smy8935@naver.com (M.S.); jin011222@naver.com (Y.K.); alseo10205@gmail.com (M.K.); jungdi@gnu.ac.kr (D.-I.J.); chokw@gnu.ac.kr (K.-W.C.)

**Keywords:** canine melanoma, feline melanoma, melanocytic tumors, diagnosis, immunohistochemistry, sentinel lymph node, surgery, radiation therapy, immunotherapy, prognosis

## Abstract

Melanocytic tumors are clinically important in veterinary oncology because their biological behavior, diagnosis, and prognosis vary substantially by species and anatomical site. Canine oral melanoma is characterized by aggressive local invasion and early metastatic spread, whereas melanocytic tumors at other canine sites and in cats show distinct clinicopathologic features that require different diagnostic and therapeutic approaches. This narrative review was based on a structured literature search of PubMed, with searches organized around epidemiology and biological behavior, diagnosis, treatment, and prognosis. A total of 1887 records were identified after removal of duplicates, and 111 publications were ultimately included following title, abstract, and full-text screening. This review summarizes current evidence on the epidemiology, molecular biology, diagnosis, clinical staging, treatment, and prognosis of canine and feline melanocytic neoplasms. Diagnostic approaches include cytology, histopathology, immunohistochemistry for amelanotic tumors, advanced imaging, sentinel lymph node mapping, and emerging molecular methods. Therapeutic options include surgery, radiation therapy, electrochemotherapy, chemotherapy, therapeutic vaccination, immune checkpoint inhibitors, and targeted therapies. Prognostic factors include tumor location and stage, histologic grade, mitotic count, nuclear atypia, pigmentation, tissue invasion, and molecular biomarkers. Surgical excision remains the cornerstone of treatment for localized disease, but effective systemic therapies for advanced melanoma remain limited. Ongoing molecular characterization, genomic profiling, and comparative oncology research may improve prognostic stratification and support more precise therapeutic strategies.

## Simple Summary

Melanoma is a malignant tumor of melanocytes that is often characterized by aggressive local invasion and a substantial potential for metastasis. As research in veterinary melanoma continues to expand, an updated synthesis of the available evidence is needed to clarify current knowledge and incorporate recent clinical and molecular advances. This review summarizes the epidemiology, biological characteristics, diagnosis, treatment, and prognosis of canine and feline melanoma, with the aim of providing a more integrated understanding of its biological and clinical characteristics and current management. By bringing together established evidence and recent developments, this review also highlights areas in which further research may improve clinical decision-making and the management of affected animals.

## 1. Review Methodology

This narrative review summarizes the current evidence on the diagnosis, treatment, and prognosis of canine and feline melanoma. A structured literature search was conducted using PubMed. The final literature search was conducted in August 2026, with no restriction on publication year. Searches were organized according to the major topics addressed in this review, including epidemiology and biological behavior, diagnosis, treatment, and prognosis.

The following Boolean search strategies were applied to PubMed:

1. Epidemiology and biological behavior (*n* = 916)

(canine or dog or feline or cat) and (melanoma or melanocytic neoplasm) and (epidemiology or incidence or prevalence or biological behavior or tumor behavior or tumor aggressiveness or local recurrence or metastasis or prognosis);

2. Diagnosis (*n* = 638)

(canine or dog or feline or cat) and (melanoma or melanocytic neoplasm) and (diagnosis or diagnostic or cytology or histopathology or immunohistochemistry or immunocytochemistry);

3. Treatment (*n* = 1,249)

(canine or dog or feline or cat) and (melanoma or melanocytic neoplasm) and (treatment or surgery or surgical resection or sentinel lymph node mapping or radiation therapy or chemotherapy or electrochemotherapy or vaccine or immunotherapy);

4. Prognosis (*n* = 185)

(canine or dog or feline or cat) and (melanoma or melanocytic neoplasm) and (prognosis or prognostic) and (staging or grading or marker or parameter).

The four search strategies retrieved a total of 1887 records after removal of duplicates, of which 111 publications were ultimately included after screening for relevance to the scope of this review. Eligible studies provided relevant information on the epidemiology, biological behavior, diagnosis, treatment, or prognosis of canine or feline melanocytic tumors. Priority was given to recent and highly cited studies, as well as older seminal studies that have been widely cited and considered foundational in the field. Canine and feline studies were prioritized, whereas relevant human melanoma studies were considered as supplementary sources when veterinary evidence was limited. Articles unrelated to the scope of this review, including those focused on non-melanocytic tumors or unrelated conditions, were excluded. Reference lists of relevant articles were additionally screened to identify potentially eligible studies not retrieved through the structured PubMed search.

## 2. Introduction

### 2.1. Pathogenesis and Molecular Biology

#### 2.1.1. Melanocyte Biology, Melanin Synthesis (Figure 1)

**Figure 1:**
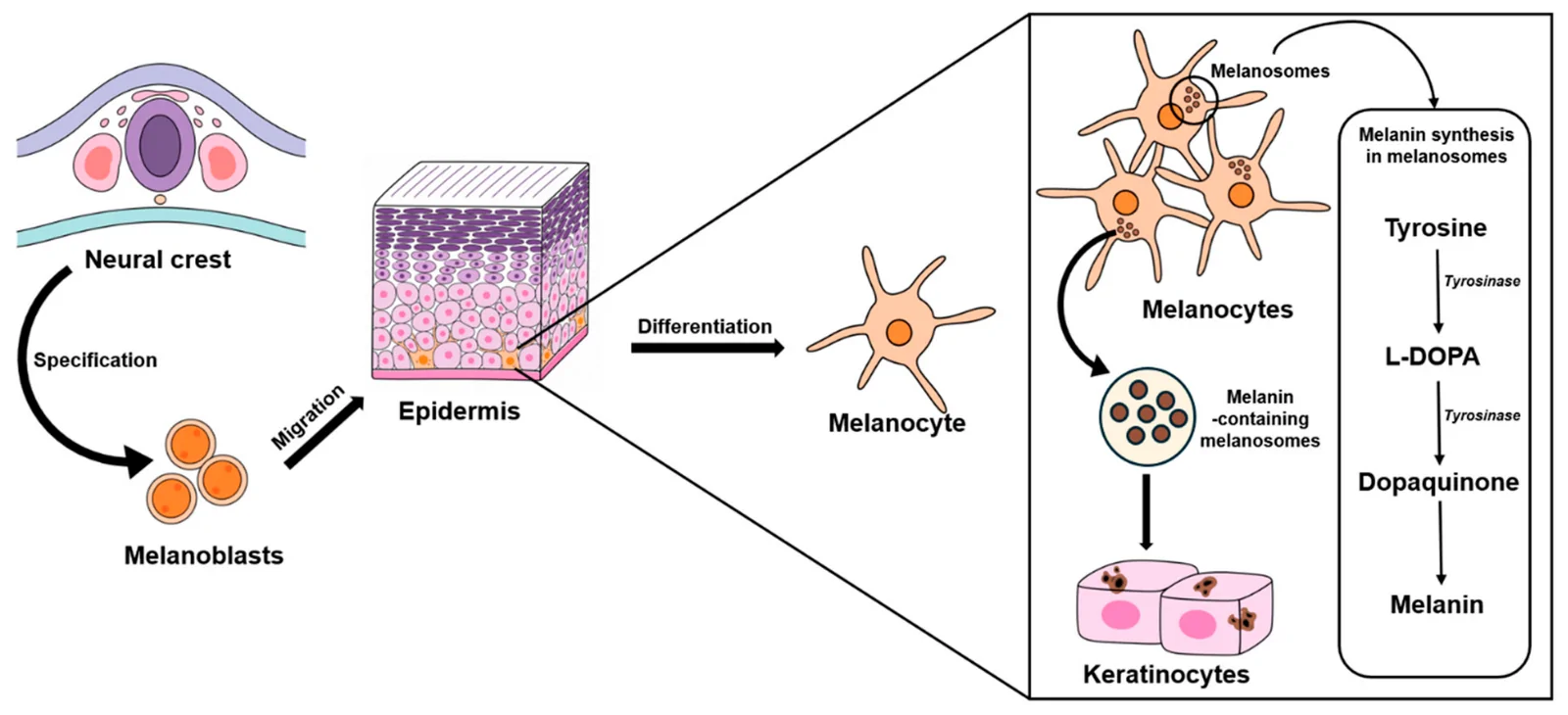
Melanocyte development, melanogenesis, and melanosome transfer. L-DOPA, L-3,4-dihydroxyphenylalanine.

Melanocytic tumors arise from melanocytes, which originate from neural crest-derived melanoblasts [1]. Melanoblasts migrate from the neural crest to the integument, where they populate the epidermis and hair follicles and differentiate into mature melanocytes or remain as melanocyte stem cells [1,2]. Alternatively, melanocytes may arise from Schwann cells or bipotential precursor cells capable of differentiating into either Schwann cells or melanoblasts that migrate along peripheral nerve sheaths [2]. Melanin is synthesized within specialized cytoplasmic organelles called melanosomes in melanocytes through a series of tyrosinase-catalyzed reactions, in which tyrosine is converted to L-3,4-dihydroxyphenylalanine (L-DOPA) and subsequently to melanin [3]. Following synthesis, melanin-containing melanosomes are transferred from melanocytes to neighboring keratinocytes through membrane-bound vesicles, resulting in the deposition of melanin within the epidermis [3,4] (Figure 1). This process is regulated by several key signaling molecules, including microphthalmia-associated transcription factor (MITF), the receptor tyrosine kinase KIT, and its ligand [5]. Although the initiating mechanisms of canine melanomagenesis remain incompletely understood, genetic alterations affecting melanocyte proliferation and survival are thought to contribute to neoplastic transformation. Several oncogenic signaling pathways associated with these processes have been identified in canine melanoma [6].

#### 2.1.2. Genomic Contributors to Tumorigenesis

Human melanoma is characterized by one of the highest mutational burdens among cancers. Recurrent driver mutations in *BRAF* (40–50%), *RAS* (15–25%), and *NF1* have been identified in human melanoma [7].

In contrast, *BRAF* mutations, which are common in human cutaneous melanoma, are rare in canine melanoma, occurring in approximately 6% of cases [8]. Nevertheless, constitutive downstream activation of ERK has been identified in both human and canine malignant melanomas, suggesting that, despite differences in driver mutations, the two species may share molecular mechanisms underlying tumor development and progression [9,10].

Transcriptomic analysis of canine oral melanoma has further demonstrated substantial molecular similarities between canine and human melanoma, with more than 80% of human melanoma-associated oncogenes shared between the two species. In addition, 429 melanoma-associated signature genes were commonly upregulated, with the JAK-STAT signaling pathway being the most significantly enriched. Furthermore, regulatory molecules such as IL6 and NGF were identified as potential regulators of melanoma-associated signaling [11]. These transcriptomic findings suggest that JAK-STAT signaling may represent a candidate molecular pathway of interest in canine melanoma. Experimental studies in canine melanoma cell lines have shown that oclacitinib can attenuate IFN-γ-induced PD-L1 expression and, in vitro, enhance radiosensitivity with suppression of STAT3 activation [12,13]. However, these findings remain preclinical and do not establish clinical antitumor efficacy.

### 2.2. Melanocytic Tumors in Dogs

#### 2.2.1. Incidence and Prevalence

Canine melanocytic tumors account for approximately 3–8% of all tumors in dogs. Of these, approximately 70% are malignant melanomas, whereas the remaining 30% are benign melanocytomas [14,15,16].

Canine melanocytic tumors occur most commonly in middle-aged to older dogs, with a peak incidence between 10 and 12 years of age [9]. No significant sex predisposition has been identified, with males and females affected at approximately equal frequencies [17]. Several breeds have been reported to be predisposed to melanoma, including the Scottish Terrier, Golden Retriever, Poodle, Dachshund, and Chow Chow [9]. The most common anatomical locations of canine melanoma are the oral cavity (62%), skin (27%), digits (6%), and subungual region (4%) [18].

#### 2.2.2. Biological Behavior and Clinical Signs

Canine melanocytic tumors exhibit distinct biological behavior and clinical manifestations depending on their anatomical site of origin (Table 1).

Oral melanocytic tumors are highly aggressive and are virtually always considered malignant [9,17,19]. Oral melanomas have a high metastatic potential, with approximately 70–90% of tumors eventually metastasizing regardless of their location within the oral cavity. The regional lymph nodes, lungs, and visceral organs, including the heart, are the most common sites of metastasis [19]. In contrast, approximately 70% of melanocytic tumors arising from the lips are malignant, representing a lower proportion than that observed in melanocytic tumors arising within the oral cavity [20]. Approximately 53% of oral melanocytic tumors arise from the labial mucosa or mandibular gingiva, whereas less common sites include the buccal mucosa, palate/pharynx, tongue, maxillary gingiva, tonsils, and other gingival locations [17]. Because these tumors are friable and prone to ulceration, clinical signs commonly include dysphagia, weight loss, halitosis, ptyalism, oral bleeding, and, occasionally, pathologic fracture of the mandible [19].

Haired skin melanocytic tumors typically present as solitary, raised, pigmented cutaneous masses without obvious signs of inflammation. Although most lesions are small, some may be large, flat, wrinkled, or multifocal. These tumors often exhibit indolent biological behavior and may persist for prolonged periods, with most lesions being biologically benign [9,21].

In contrast to melanocytic tumors arising in haired skin, those at specialized cutaneous sites may behave more aggressively. Digital melanocytic tumors have substantially greater metastatic potential, with metastasis reported in approximately 50% of cases [22]. Similarly, approximately 40% of scrotal melanocytic tumors are malignant [23]. Digital melanocytic tumors occur more frequently in the forelimbs (62.8%) than in the hindlimbs (37.2%) [22]. Subungual lesions typically present as swollen, painful distal digits or as non-healing lesions adjacent to the nail. Affected dogs commonly exhibit lameness or excessive licking of the affected digit [24]. Bone lysis of the affected distal phalanx is frequently observed [22]. In the scrotum, melanocytomas are typically solitary, small, pigmented, firm, and freely movable over the underlying tissues. In contrast, malignant melanomas are generally rapidly growing, pigmented masses that are frequently ulcerated [23].

### 2.3. Melanocytic Tumors in Cats

In cats, melanocytic tumors are relatively uncommon, accounting for less than 1% of all neoplastic diagnoses [25]. Reported median age at diagnosis ranges from 11 to 13 years, although melanocytic tumors of the pinna have been reported in younger cats, with a median age of 7 years. As in dogs, no significant sex predisposition has been identified, with males and females affected at approximately equal frequencies [26,27,28]. Ocular melanocytic tumors, particularly those involving the iris, are most common; cutaneous tumors most often arise on the pinnae, whereas oral melanocytic tumors are relatively rare. Melanocytomas are exceptionally uncommon in cats [29,30].

The clinical manifestations of ocular melanoma vary with the ocular structure involved and lesion location. Clinical signs may include a visible intraocular or periocular mass, glaucoma, hyphema, anterior uveitis, epiphora, conjunctival hyperemia, mucopurulent ocular discharge, and/or protrusion of the third eyelid [31].

## 3. Diagnosis

Melanoma exhibits variable clinical behavior and is generally considered an aggressive neoplasm. Accurate diagnosis is therefore essential before comprehensive tumor assessment for prognosis and treatment planning.

In heavily pigmented tumors, a melanoma may initially be suspected by cytologic evaluation of fine-needle aspiration samples. Melanomas can exhibit diverse cytomorphologic features, including epithelioid, spindle-shaped, and round-cell forms. However, approximately one-third of canine melanomas are amelanotic and may lack visible intracytoplasmic melanin granules. In such cases, additional diagnostic testing is required to distinguish melanoma from other neoplasms in the differential diagnosis. Melanocytic origin may then be established by immunocytochemistry on cytologic specimens or by histopathologic examination with immunohistochemistry. Subsequent evaluation of relevant prognostic factors may further aid estimation of clinical outcome.

Once melanocytic origin has been established, biological behavior should be assessed by integrating histologic grade with tumor location, size, degree of local invasion, and metastatic status during clinical staging. This integrated approach supports prognostic assessment and selection of an appropriate therapeutic strategy.

### 3.1. Cytology

**Figure 2:**
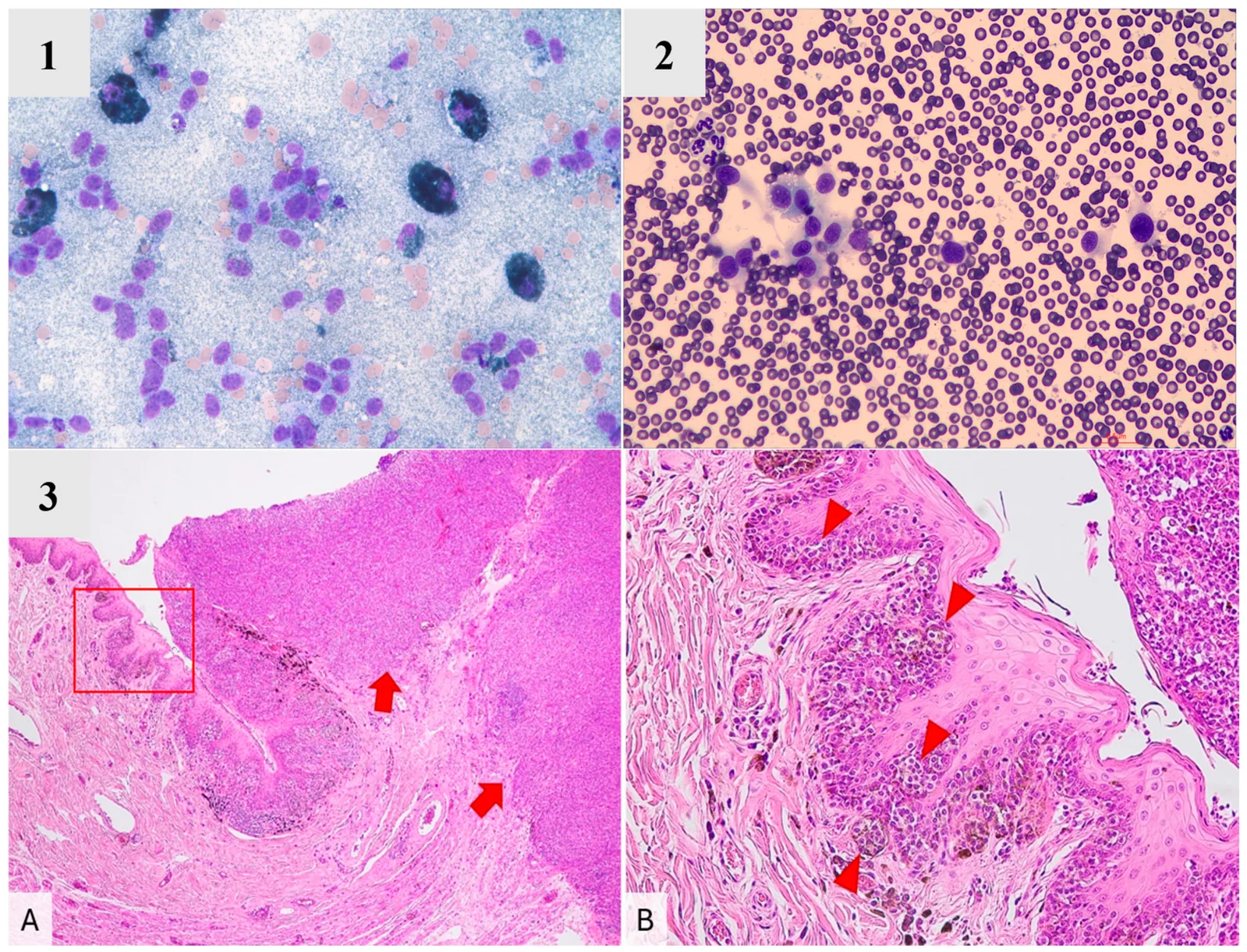
Fine-needle aspiration cytology (**1**,**2**) and histologic section (**3**) of canine melanoma. (**1**) Round-to-oval neoplastic melanocytes containing abundant melanin pigment. (**2**) Spindle-shaped neoplastic melanocytes exhibiting marked anisokaryosis, multiple prominent nucleoli, and a lack of cytoplasmic pigment. (**3**) Histopathologic features of canine oral melanoma showing junctional activity. (**3A**) Low-magnification view of the peripheral region of a malignant melanoma; arrows indicate the tumor mass. (**3B**) Higher-magnification view of the boxed area in (**A**), demonstrating multiple nests of neoplastic cells scattered within the mucosal epithelium (arrowheads). Source: FUJIFILM VET Systems Co., Ltd., “Pathology and Cytology Diagnostician Column No. 2: Oral Malignant Melanoma in Dogs,” Figure 2. Available at: https://www.fujifilm.com/jp/ja/healthcare/veterinary/column/ca-ve-k9-oral-melanoma (accessed on 2 August 2026). Reproduced with permission from FUJIFILM VET Systems Co., Ltd. FUJIFILM Holdings Corporation, Tokyo, Japan [40].

Pigmented melanoma may be readily identified by cytologic examination because neoplastic cells characteristically contain intracytoplasmic melanin [30,31]. Well-differentiated neoplastic melanocytes typically exfoliate individually or in small clusters and exhibit a round to polygonal morphology. These cells contain abundant melanin pigment within a relatively small amount of cytoplasm and have round-to-oval nuclei with a single large, prominent nucleolus [9,32,33,34,35,36,37] (Figure 2(1)). Cytologic examination has been reported to have high sensitivity and specificity for the diagnosis of pigmented melanomas [32,38]. However, the degree of pigmentation may vary according to the level of tumor differentiation, limiting the diagnostic utility of cytology alone in poorly pigmented or amelanotic tumors [35,36].

Poorly differentiated neoplastic melanocytes typically exhibit marked cytologic atypia, including pronounced anisocytosis, multinucleation, a high nuclear-to-cytoplasmic ratio, multiple nucleoli, and cytoplasmic vacuolation. Because these cells may contain minimal or no visible melanin pigment (Figure 2(2)), they may be difficult to distinguish cytologically from other neoplasms, including pigmented basal cell tumors, peripheral nerve sheath tumors, squamous cell carcinomas, and fibrosarcomas [9,34,35,36,37]. Accordingly, a melanoma should remain in the differential diagnosis when poorly differentiated cells with these cytologic features are identified, regardless of the apparent cell type or tissue of origin [9,34,35,36,37].

Accordingly, when cytologic findings are inconclusive, ancillary diagnostic testing, such as histopathologic examination or immunocytochemistry, is indicated to establish a definitive diagnosis [28,34]. In canine melanoma, neoplastic cells generally demonstrate strong immunoreactivity to anti-Melan-A antibodies [39]. However, because Melan-A immunoreactivity may occasionally be observed in certain epithelial neoplasms, its interpretation should be supported by a panel of additional markers [39]. The combined use of anti-cytokeratin, which supports epithelial differentiation, and anti-vimentin, which supports mesenchymal differentiation, can improve diagnostic sensitivity and specificity. Within this immunophenotypic context, an amelanotic melanocytic neoplasm is typically characterized by positive immunoreactivity for Melan-A and vimentin and negative immunoreactivity for cytokeratin [34,39].

Importantly, abundant pigmentation and a well-differentiated cytologic appearance do not necessarily indicate indolent biological behavior, because some well-differentiated melanocytic neoplasms may still behave aggressively. Tumor location, in particular, is recognized as an important determinant of biological behavior. Therefore, assessment should extend beyond cytology to include anatomical location and clinical stage, not only when melanocytic origin remains uncertain but also when a well-differentiated, heavily pigmented tumor is identified cytologically [35].

### 3.2. Histopathology

Histopathologic examination is essential for diagnosing melanoma because it permits evaluation of morphologic features not fully assessable by cytology. Beyond individual cellular features, histopathology enables assessment of tissue architecture, growth pattern, and the spatial relationship between the tumor and adjacent structures [32,34,39].

When obtaining a biopsy specimen, preservation of the overlying epithelium and the epithelial–stromal interface is particularly important. Neoplastic melanocytes may exhibit junctional activity, intraepithelial nesting, and lentiginous spread along the epidermal–dermal or mucosal–submucosal junction. These architectural features provide important diagnostic evidence supporting melanocytic origin [28,32,41].

Smedley et al. [32] emphasize that intact epithelium should be included in the biopsy specimen even when the central portion of the lesion is ulcerated. Because melanocytic neoplasms may extend laterally through lentiginous spread, neoplastic cells and diagnostically relevant junctional changes may persist within intact epithelium adjacent to the ulcerated lesion (Figure 2(3)). Preservation and adequate sampling of this peripheral epithelium may therefore improve diagnostic accuracy. In addition, when biopsying an oral melanoma, the approach should be carefully planned to minimize unnecessary tumor disruption and the potential risk of tumor-cell seeding through uninvolved mucosa [28].

As in cytologic specimens, melanomas may show considerable morphologic variability. Neoplastic cells may be epithelioid or spindle-shaped, with a mixed epithelioid and spindle-cell pattern being the most common morphology [17]. Poorly differentiated melanomas may also show less common morphologic variants, including balloon cells, signet-ring cells, round cells, and angiomatoid cells. These tumors often exhibit marked anisocytosis and anisokaryosis, multinucleation, variable chromatin patterns, and reduced or absent pigmentation, mirroring the atypical cellular features observed cytologically [32,41].

However, when diagnostically relevant architectural features, such as junctional activity, are absent or poorly preserved and the constituent cells are poorly differentiated, histopathologic examination alone may be insufficient to distinguish a melanoma from poorly differentiated carcinoma or soft tissue spindle-cell sarcoma. In such cases, immunohistochemistry using an appropriate antibody panel is required to support melanocytic differentiation and exclude alternative diagnoses.

### 3.3. Immunohistochemistry

Given species-dependent variability in the sensitivity and specificity of antibodies used for immunohistochemistry (IHC), a panel of melanocyte-targeting antibodies generally provides greater diagnostic accuracy than a single antibody [32,39].

In melanomas, intraepithelial neoplastic cells commonly represent a more differentiated tumor-cell population and may exhibit stronger expression of melanocytic markers. Accordingly, biopsy specimens from non-ulcerated regions with intact epithelium are more likely to contain diagnostically informative cells and may improve the accuracy of immunohistochemical diagnosis [32,39].

Melanocytic markers, including Melan-A, PNL-2, HMB-45, S-100, microphthalmia-associated transcription factor (MITF), tyrosinase-related protein 1 (TRP-1), and TRP-2, can facilitate the diagnosis of nonpigmented (amelanotic) melanoma [42]. Although S-100 and MITF exhibit relatively low specificity, they are generally considered highly sensitive markers of melanocytic differentiation. An immunohistochemical cocktail comprising Melan-A, PNL-2, TRP-1, and TRP-2 demonstrated 100% specificity against soft tissue spindle cell sarcomas (10/10; 95% CI, 69.2–100%) and 93.9% sensitivity for canine oral amelanotic melanocytic tumors (46/49; 95% CI, 83.1–98.7%) [34,43]. Both confidence intervals were calculated from the reported numerators and denominators using the exact binomial method. However, the sensitivity of this marker combination may be reduced in spindle-cell variants. In such cases, SOX-10 may serve as a useful complementary marker because of its high sensitivity for melanocytic differentiation, although its comparatively lower specificity requires interpretation in conjunction with tumor morphology and the results of other immunohistochemical markers [37].

In cases where the biopsy specimen lacks intact epithelium or diagnostically informative junctional features, molecular profiling may serve as a valuable adjunct to conventional histopathologic and immunohistochemical evaluation. In particular, comparative messenger RNA expression analysis of *TYR*, *CD34*, and *CALD1* may facilitate the differential diagnosis of oral soft-tissue sarcoma and spindle-cell oral amelanotic melanoma [37].

### 3.4. Emerging Molecular Diagnostic Approaches

Recent studies have evaluated molecular approaches both as adjuncts to conventional diagnosis and as tools for longitudinal disease monitoring in canine malignant melanoma.

Liquid biopsy-based approaches, including analysis of circulating cell-free DNA (cfDNA) and the DNA integrity index (DII), have been investigated as minimally invasive biomarkers [44,45]. cfDNA consists of extracellular DNA fragments released into the circulation from normal and neoplastic cells and may partly reflect tumor burden, whereas DII provides an indirect measure of cfDNA fragmentation patterns [44,45]. In one study, dogs with oral melanoma had significantly lower DII values than healthy controls, supporting its potential utility as an adjunctive diagnostic biomarker. Longitudinal changes in cfDNA concentrations and DII values were also associated with disease recurrence and progression, supporting potential use in monitoring disease status and treatment response [44].

Genomic and transcriptomic profiling has also contributed to molecular characterization of canine oral melanoma and identification of candidate biomarkers and therapeutic targets. Integrated genomic and transcriptomic analyses showed that recurrent amplification of the CFA30 region encompassing *TRPM7* was associated with increased gene expression and enhanced tumor cell proliferation, suggesting potential utility for molecular subclassification and therapeutic target identification; however, these findings remain at the discovery stage and require independent confirmation before clinical translation [46]. In addition, an integrative in silico comparison of canine and human melanoma datasets identified 60 genes commonly altered across both species. Subsequent protein–protein interaction network analysis identified *RPL8*, *POLR2B*, *FBXO32*, and *SOCS5* as highly connected hub genes, illustrating the potential of comparative genomic approaches to identify candidate molecular biomarkers; however, their clinical diagnostic and prognostic utility remains to be validated [42].

## 4. Clinical Staging

### 4.1. Sentinel Lymph Node (SLN) Mapping and Biopsy

Evaluation of the sentinel lymph node (SLN) serves as a vital diagnostic tool to improve staging accuracy and guide clinical decision-making, although a direct survival benefit of prophylactic nodal removal remains unproven. Regional lymph node extirpation is primarily indicated when metastasis is suspected or confirmed. The SLN is defined as the first lymph node within the lymphatic basin that receives drainage directly from the primary tumor [46,47]. In human oncology, when the SLN is free of metastasis, the remaining lymph nodes are also negative in more than 90% of cases. Similarly, a study of canine oral melanoma reported a 0% false-negative rate for SLN biopsy, supporting its potential reliability for detecting metastatic spread [48].

Among mapping techniques, indirect computed tomography lymphangiography (ICTL) is widely used. This technique involves peritumoral injection of a contrast agent followed by CT to trace lymphatic drainage pathways three-dimensionally and identify the SLN [48,49,50,51]. Near-infrared fluorescence lymphography using indocyanine green involves injection of a fluorescent dye and intraoperative visualization of the SLN with a specialized near-infrared camera; it is frequently combined with ICTL to improve diagnostic accuracy [52,53].

Alternatively, methylene blue staining enables intraoperative visual localization of the lymph node, whereas lymphoscintigraphy uses a radiotracer and an intraoperative gamma probe to detect radioactive nodes [54,55,56,57].

Preoperative contrast-enhanced ultrasound uses microbubble contrast agents to monitor real-time lymphatic drainage [58], whereas indirect radiographic lymphography uses standard radiography and contrast media rather than CT [59]. Ultimately, while evaluation and biopsy of draining lymph nodes or the corresponding lymphatic basin provide critical prognostic and staging information for canine oral and digital melanomas, elective lymph node extirpation should be recommended with caution, as a definitive therapeutic or survival benefit has not yet been established.

### 4.2. Clinical Staging System

Clinical stage is assigned using the tumor–node–metastasis (TNM) system, which incorporates maximum primary tumor diameter (T), regional lymph node metastasis (N), and distant metastasis (M) (Table 2). Maximum primary tumor diameter may be measured with calipers or computed tomography (CT). The extent of local invasion, however, is not formally included as a major determinant in the current staging system [9,28,34].

Melanoma exhibits a marked propensity for lymphatic dissemination, and accurate assessment of regional lymph node status is clinically important because nodal metastasis may influence prognosis. In canine oral melanoma, the mandibular lymph nodes are commonly affected; however, lymphatic drainage within the head is complex and may involve contralateral or otherwise unexpected lymph nodes. Sentinel lymph node mapping is therefore important for accurately identifying and evaluating the lymph nodes that directly drain the primary tumor [9]. Histopathologic examination of excised lymph nodes remains the gold standard for confirming nodal metastasis. Although fine-needle aspiration is convenient and minimally invasive, its agreement with histopathology is limited [60]. When metastatic cells are not readily identifiable on routine cytologic examination, immunocytochemistry using Melan-A and PNL-2 may improve detection of neoplastic melanocytes and help distinguish them from melanophages that may normally be present within lymph nodes [32].

Distant metastasis is primarily assessed using three-view thoracic radiography or thoracic CT. Although less frequently reported, involvement of abdominal lymph nodes and intra-abdominal organs should also be considered during clinical staging. Distant metastasis is consistently associated with an unfavorable prognosis, whereas the prognostic significance of regional lymph node metastasis appears to be less pronounced [9,32,60].

A recent study by Pérez-Santana et al. highlighted the limited ability of the conventional clinical staging system to discriminate median survival times among affected dogs, partly due to methodological limitations, such as limited standardization of histopathologic characteristics across study populations and the lack of adjustment of tumor size for patient body size. These issues highlight the need for further studies using more standardized methodologies [61]. The authors also described a previously proposed modified staging system incorporating tumor volume, anatomical location, and mitotic index, noting that each of these factors has been reported to have prognostic relevance in multiple studies [41,61].

## 5. Therapeutic Approach

For dogs and cats without evidence of distant metastasis, treatment is primarily directed toward local tumor control. Surgical excision remains the cornerstone of therapy and the gold standard for localized melanoma. Radiation therapy (RT) also has an important role, providing palliation for unresectable tumors and postoperative local control when histologic margins are incomplete.

For tumors that cannot be completely excised because of their location or extent of invasion, alternative local therapies have been investigated. Electrochemotherapy (ECT) has emerged as a potential option for unresectable disease.

Because distant metastasis is a major cause of treatment failure, systemic therapy may be used as an adjuvant treatment for animals at high risk of metastatic disease or as palliation for animals with advanced or unresectable tumors. Current systemic approaches include conventional cytotoxic chemotherapy, therapeutic vaccination, immune checkpoint inhibitors, and targeted therapies. Although several approaches have shown promising results, robust prospective clinical evidence remains limited, and further studies are needed to establish efficacy and define their optimal roles in melanoma management.

### 5.1. Local Treatment

#### 5.1.1. Surgical Treatment

Selection of the surgical approach depends largely on tumor location. Although most cutaneous melanomas can be managed by local excision, oral lesions and tumors at other specialized sites generally require more extensive resection, such as mandibulectomy, to achieve appropriate surgical margins [62].

##### Standardization of Surgical Margin Evaluation Criteria and Techniques

Wide surgical margins of approximately 1 cm for benign cutaneous melanocytic tumors and 2–3 cm for malignant melanoma are generally recommended when anatomically feasible [26]. However, interpretation of surgical margins remains inconsistent because veterinary oncology lacks universally accepted histopathologic criteria for complete tumor excision. To address this limitation, Liptak proposed the residual tumor (R) classification as a standardized framework for margin reporting. The R classification categorizes resections as R0, R1, or R2: R0 indicates no neoplastic cells at the inked surgical margin, whereas R1 and R2 indicate microscopic and macroscopic residual disease, respectively.

Interpretation of histologic surgical margins is further complicated by tissue shrinkage after excision and formalin fixation, which may lead to underestimation of the margin achieved at surgery. Reported specimen shrinkage in canine skin ranges from approximately 15% to 42%. The R classification therefore provides a standardized framework for reporting residual disease, improving consistency in pathologic interpretation and facilitating comparison of surgical outcomes among studies [31].

##### Oral Melanoma

Canine oral melanoma is a highly aggressive neoplasm characterized by local invasion and high metastatic potential. Consequently, conservative surgical approaches are generally discouraged because limited excision is associated with local recurrence rates exceeding 70% and a median survival time of 3 to 4 months. Wide or radical surgical excision with adequate bone and soft tissue margins, achieved through mandibulectomy or maxillectomy, remains the cornerstone of treatment [9] and is often combined with regional lymphadenectomy for accurate staging and regional disease control [62].

Across large retrospective studies, curative-intent surgery achieved histologically complete margins in 72.9–79.3% of dogs, with low local recurrence rates (8.3–17%) and median survival times ranging from 354 to 723 days [35,63]. Despite these favorable outcomes, the optimal anatomical extent of bone resection remains uncertain. Studies found no significant differences in local recurrence or metastatic rates between aggressive en bloc resections, such as unilateral mandibulectomies, and more conservative partial mandibulectomies [64].

Furthermore, the prognostic value of histologically clean margins remains uncertain. Although early studies suggested a survival benefit, recent large-scale retrospective investigations did not identify an association between tumor-free margins and longer median survival times, noting that local recurrence frequently occurred even after histologically complete excision [64,65,66]. This discrepancy may reflect two factors: first, conventional histopathology assesses less than 1% of the total margin surface area, leading to false-negative results and undetected microscopic disease; second, overall prognosis may be influenced more by occult systemic micrometastasis than by local disease control alone [26,31]. Because systemic metastasis is common, multimodal treatment incorporating adjuvant therapies such as chemotherapy [67], radiation therapy, and xenogeneic DNA vaccination [68] is frequently recommended.

Because canine oral melanoma is highly metastatic, thorough regional lymph node assessment is important alongside primary tumor resection. Noninvasive staging may be insufficient; up to 40% of normal-sized, nonpalpable lymph nodes harbor occult micrometastases [69], and computed tomography sensitivity for predicting cervical metastasis varies widely from 12% to 83% [70,71]. Furthermore, oral lymphatic drainage is unpredictable, with contralateral metastasis occurring in up to 62% of cases and 26.7% to 45.5% of regional metastases involving nodes other than the superficial mandibular lymph nodes (MLNs). Notably, approximately 6% of canine oral melanoma cases exhibit isolated metastasis to the medial retropharyngeal lymph nodes (MRLNs) without concurrent MLN involvement. Relying solely on MLN extirpation therefore risks significant understaging [48]. To optimize staging accuracy, bilateral extirpation or evaluation of both MLNs and MRLNs has been advocated; however, elective neck dissection in clinically negative (cN0) necks should be recommended with caution, as a definitive therapeutic or survival benefit of prophylactic lymphadenectomy remains to be established [72].

##### Cutaneous, Digital, and Footpad Melanoma

The clinical behavior of canine non-oral melanocytic tumors is largely determined by anatomical site. Melanocytic tumors arising from haired skin generally exhibit benign biological behavior, and local excision is curative in most cases. In a retrospective study of 87 dogs, Laver et al. (2018) reported outcomes after surgical resection alone, with low rates of local recurrence (8%) and subsequent metastasis (21.8%) [73,74,75].

Conversely, canine melanocytic tumors of the digits and footpads can behave aggressively and carry a guarded long-term prognosis. In dogs without detectable regional or distant metastasis, curative-intent local control is primarily achieved by digit amputation. For subungual melanomas, radical amputation at the metacarpophalangeal or metatarsophalangeal joint is recommended to achieve tumor-free margins [26].

##### Ocular Melanoma

Ophthalmic melanocytic tumors in dogs and cats exhibit highly variable biological behavior depending on species and anatomical location. Intraocular melanomas in both species are frequently diagnosed at advanced stages, limiting therapeutic options [76]. Enucleation is therefore frequently required, although globe-preserving strategies, including local surgical excision (LSE), cryotherapy, radiation therapy, photodynamic therapy, and laser ablation, can be considered for localized, noninvasive lesions.

Canine limbal melanocytic tumors are usually benign and slow-growing but may progressively invade the cornea and sclera [77]. Standard management involves sclerectomy, occasionally combined with lamellar keratectomy [78,79]. Although adjunctive strontium-90 beta plesiotherapy can provide high local control, 20% of dogs experience severe late-onset complications, such as deep scleral thinning, lipid keratopathy, or globe perforation [78,80]. Diode laser photocoagulation may reduce radiation-associated morbidity, whereas Nd:YAG laser therapy carries a risk of corneal degeneration [77]. Alternatively, adjunctive 1,1,1,2-tetrafluoroethane cryotherapy performed with partial lamellar resection and grafting was associated with a reported 100% success rate and no recurrence in all 14 treated eyes over a median follow-up of 2.1 years (range, 6 months to 8.5 years), with transient early complications (uveitis, corneal ulceration, and granulation tissue) and late corneal lipidosis as the primary sequelae [81].

Conjunctival melanocytic tumors are primarily managed by complete surgical excision with adjunctive cryotherapy. Tumors involving the third eyelid may necessitate partial or complete third-eyelid excision, whereas extensive or invasive lesions are generally managed by enucleation [80]. Canine uveal melanocytomas present as raised, pigmented masses within the iris or ciliary body. A study evaluating globe-preserving LSE in five dogs reported no recurrence at 3 months postoperatively, although common transient complications included hyphema, uveitis, and diffuse corneal edema [82].

##### Melanocytoma

Unlike melanomas, melanocytomas are benign, slow-growing pigmented tumors that most commonly arise in the skin and are associated with a substantially more favorable prognosis. Complete surgical excision is generally curative, and favorable long-term outcomes are expected even in dogs with multicentric disease [15]. Supporting these findings, Gillard et al. (2014) reported that dogs with melanocytoma had a median survival time of 1463 days, during which no local recurrence or tumor-related mortality was documented [16].

#### 5.1.2. Radiation Therapy

##### Clinical Efficacy and General Outcomes of Radiation Therapy

RT is an established local treatment that targets proliferating cells and is generally more effective against microscopic than macroscopic disease. In canine melanoma, RT may be used as postoperative adjuvant therapy or as a primary palliative modality to reduce tumor burden and alleviate pain, with a generally low incidence of severe adverse effects. Reported response rates range from 25% to 44% for partial response (PR) and 41% to 69% for complete response (CR) [19]. However, in canine oral melanoma, long-term outcomes after local curative-intent treatment are limited by aggressive biological behavior, with 44% to 58% of dogs eventually experiencing distant metastatic failure [9]. Overall response rates (ORRs) for canine oral melanoma are estimated at 75–85% when CR and PR are combined, with reported median survival times (MSTs) of 230 to 363 days [83,84,85]. Importantly, a clear distinction must be made between local therapeutic response (ORR, local tumor control) and long-term clinical benefits, such as progression-free survival (PFS) and overall survival (OS). While RT consistently achieves high local objective response rates and effective local control, a high ORR does not necessarily imply improved long-term survival. Similarly, Cunha et al. reported an ORR of 93% after orthovoltage RT (29% CR and 64% PR), while survival remained stage-dependent, with MST declining from 390 days in stage I to 90 days in stage IV [86].

##### Efficacy and Limitations of Hypofractionated RT Protocols

Biologically, estimates derived primarily from human melanoma and experimental models indicate a low α/β ratio (0.5–2.5 Gy), similar to that of late-responding normal tissues, which renders melanoma cells particularly susceptible to higher radiation doses per fraction. Based on this rationale, hypofractionated protocols (e.g., 3 to 6 fractions administered once or twice weekly) are commonly used in veterinary melanoma radiation therapy, even though direct species-specific α/β values for canine and feline melanomas remain less defined [87,88].

Bateman et al. evaluated an outpatient protocol consisting of three 8-Gy fractions delivered on days 0, 7, and 21 (24 Gy total), achieving an ORR of 83%, including CR and PR rates of 53% and 30%, respectively, in 17 dogs. Acute toxicity was mild and limited to the irradiated skin, and no late radiation-associated adverse effects were reported. This protocol can reduce the physiologic and financial burden of repeated anesthesia and was associated with low-grade, self-limiting acute toxicities that resolved within 3 months without chronic damage [83]. Proulx et al. (*n* = 140) reported no significant differences in objective response, time to first event, or survival among 30 Gy (10 Gy × 3), 36 Gy (9 Gy × 4), and conventional fractionated RT exceeding 45 Gy [9].

Conversely, limited clinical data suggest that feline oral melanomas may be relatively less radiosensitive than canine tumors. In a small study of five cats evaluated by Farrelly et al. using the same 24-Gy protocol, a lower ORR of 60% (20% CR) was reported, suggesting that higher radiation doses or modified fractionation schemes may warrant further investigation in cats [89]. Although these findings suggest that treatment response may differ between feline and canine oral melanomas, the very limited feline data do not allow conclusions regarding species-specific differences in radiosensitivity.

##### RT-Related Prognostic Factors

Multivariable analysis by Proulx et al. identified macroscopic tumor volume and radiographic bone lysis as important prognostic factors for both survival time and time to first event. Dogs with no risk factors (microscopic tumor burden and absence of bone lysis) had a median survival time of 21 months, whereas dogs with all risk factors had an MST of 3 months [9].

#### 5.1.3. Electrochemotherapy (ECT)

ECT has been investigated as a treatment option in a limited number of studies for unresectable oral melanoma and has been proposed as a local treatment modality [90,91,92].

In a longitudinal prospective study, Tellado et al. evaluated ECT as a sole treatment in 67 dogs with oral melanoma, without concurrent surgery or radiation therapy. According to the Response Evaluation Criteria in Solid Tumours (RECIST), objective response (OR) rates were 100% for stage I, 89.5% for stage II, 57.7% for stage III, and 36.4% for stage IV. The median survival times following treatment were 16.5 months for stage I, 9.0 months for stage II, 7.5 months for stage III, and 4.5 months for stage IV. The authors concluded that ECT may be an adequate local treatment modality for canine oral melanoma, particularly for lesions up to 4 cm in diameter (i.e., stage I and II disease) without bone invasion [90].

In a retrospective study evaluating the addition of immunotherapy via gene electrotransfer (GET) with interleukin (IL)-2 and IL-12 to ECT in 30 dogs with stage III or IV oral melanoma, the ECT + GET group (*n* = 10) and the ECT-alone group (*n* = 20) demonstrated comparable local response rates and overall survival times. However, progression-free survival was significantly longer in the ECT + GET group, suggesting that ECT combined with IL-2 and IL-12 GET may represent a potential therapeutic option for dogs with unresectable stage III or IV oral melanoma [91].

In a prospective study, Maglietti et al. evaluated ECT using combined intravenous and intratumoral bleomycin administration in 22 dogs. According to the WHO criteria for tumor response, the combination group achieved an OR rate of 91%, including a CR rate of 54% and a PR rate of 36%, whereas the control group receiving intravenous bleomycin alone achieved an OR rate of 19%, with no CR and a PR rate of 19%. The higher response in the combination group may reflect improved intratumoral drug distribution in tumors with abnormal vasculature, which can limit homogeneous drug delivery after systemic administration alone. However, long-term outcomes, including disease-free interval and median survival time, were not reported. Tumor response was assessed at 30 days after treatment, with clinical follow-up limited to 60 days. Thus, the study provides limited information on long-term tumor recurrence and overall survival [92].

#### 5.1.4. Cisplatin Implantation

In a prospective study, Kitchell et al. evaluated intralesional cisplatin implantation in 20 dogs with oral melanoma. Tumor volume was reduced by more than 50% in 14 dogs (70%), of which 11 (55%) achieved a CR [93]. However, no subsequent studies have evaluated this approach, and its clinical role therefore remains uncertain.

### 5.2. Systemic Treatment

Surgery and RT remain the primary local treatment modalities for melanoma. When definitive local treatment is not feasible, systemic or other alternative therapies may be considered. Adjuvant systemic therapy may also be considered for dogs at high risk of metastasis, including those with oral, digital, or footpad melanoma and other melanoma [9,26].

#### 5.2.1. Cytotoxic Chemotherapy

##### Adjuvant Cytotoxic Chemotherapy

The primary objectives of combining chemotherapy with RT are to enhance tumor-cell radiosensitivity and reduce the risk of metastatic disease [9]. However, most studies have not demonstrated a significant benefit from adding chemotherapy to RT compared with RT alone [64]. Murphy et al. (2005) found no significant differences in survival time or metastatic rate between dogs receiving RT alone and those receiving RT plus carboplatin [94]. Similarly, in a large retrospective study by Proulx et al. involving 140 dogs with canine oral melanoma, 80 dogs (57%) received RT concurrently with systemic chemotherapy. Chemotherapeutic agents included carboplatin (60 dogs: median dose 225 mg/m^2^ every 3 weeks, median 4 doses), cisplatin (3 dogs: 40 mg/m^2^ for 2 to 3 doses), and melphalan (17 dogs: median dose 0.5 mg/kg every 4 weeks, median 2 doses) [9]. The analysis found that systemic chemotherapy was not associated with improvement in local response, tumor regrowth, distant metastasis, time to first event, or overall survival. A prospective study by Cancedda et al. compared coarsely fractionated RT alone (*n* = 15) with RT followed by temozolomide (TMZ; *n* = 12) in dogs with measurable oral melanoma. Although the overall response rate was similar between the groups (86.7% vs. 81.1%), adjuvant TMZ significantly prolonged median time to progression (205 vs. 110 days; *p* = 0.046) and reduced the overall failure rate (50% vs. 100%; *p* = 0.001). Median overall survival was longer in the TMZ group (401 vs. 192 days), although the difference was not statistically significant (*p* = 0.093). Notably, brain metastases were observed only in the RT-alone group, consistent with the ability of TMZ to penetrate the blood–brain barrier. Treatment was well tolerated, with no severe adverse events or hematologic toxicity [85]. These findings suggest that adjuvant TMZ may delay disease progression while maintaining a favorable safety profile.

Previous studies have shown that adjuvant chemotherapy following surgery does not provide a significant survival benefit compared with surgery alone in dogs with unresectable or progressive tumors [64]. However, in a retrospective study by Milevoj et al. evaluating metronomic chemotherapy (MC) with cyclophosphamide in 5 dogs with oral melanoma, 1 dog that received MC as adjuvant therapy following surgical excision remained progression-free for more than 529 days, while the other 4 dogs progressed within 30 days. These preliminary findings suggest that the combination of surgery and MC may prolong progression-free survival [95]. However, the clinical benefit of MC in the curative treatment of oral melanoma has not yet been well established. Therefore, dogs with oral melanoma or other malignant oral neoplasms should receive definitive local therapy whenever feasible, with adjuvant maximum tolerated dose chemotherapy considered when indicated [96].

##### Cytotoxic Chemotherapy Alone

Xia et al. retrospectively evaluated cytotoxic chemotherapy as sole treatment in dogs with oral melanoma that did not undergo curative-intent surgery or radiation therapy. 11 dogs received either maximum tolerated dose carboplatin (250 to 300 mg/m^2^; *n* = 7) or MC with chlorambucil or cyclophosphamide (*n* = 4) [97]. The median overall survival time was 181 days (range, 50 to 960 days), suggesting that cytotoxic chemotherapy may be a treatment option when definitive local treatment is not feasible [97].

#### 5.2.2. Immunotherapy

The tumor microenvironment is increasingly recognized as relevant to melanoma. In a prospective study, Yasumaru et al. analyzed tumor-infiltrating lymphocytes (TILs) in tissue samples from 45 dogs with oral melanoma [98]. Among 48 tumor samples classified as showing brisk, non-brisk, or absent TIL patterns, 28 (58%) were brisk, 10 (21%) non-brisk, and 10 (21%) absent. Dogs with longer survival had higher TIL scores, were more likely to show brisk or non-brisk patterns, and had a higher frequency of tumor-infiltrating CD8^+^ T lymphocytes [98].

##### Vaccination

Oncept™ (Merial, Duluth, GA, USA), a bacterial plasmid DNA vaccine encoding the xenogeneic human tyrosinase (huTYR) tumor-associated antigen, is the most extensively studied therapeutic vaccine for canine oral melanoma [64]. Tyrosinase is a highly conserved enzyme involved in melanin biosynthesis across mammalian species. It catalyzes the rate-limiting step of melanin production through the oxidation of L-3,4-dihydroxyphenylalanine (L-DOPA) [99]. Oncept is designed to elicit an immune response against tyrosinase-expressing melanoma cells, thereby promoting tumor cell elimination. However, the available evidence to date has not demonstrated a definitive improvement in disease-free interval or overall survival with Oncept. In particular, concerns remain regarding its efficacy in dogs with amelanotic melanoma, which lacks visible melanin pigmentation and may exhibit reduced tyrosinase expression. Nevertheless, Oncept remains a therapeutic option for canine oral melanoma despite the limited evidence supporting its clinical efficacy [99].

In a prospective study, Finocchiaro et al. evaluated cytokine-enhanced gene vaccination in 337 dogs with melanoma over a 7-year period. Patients were provisionally allocated according to the extent of surgery, either complete surgery (CS) or partial surgery (PS), and the local adjuvant treatment administered to the surgical site, either a lipoplex carrying the canine interferon-β (cIFNβ) gene plus bleomycin (CTIF/B) or 5-fluorouracil (CT5FU). Accordingly, four adjuvant treatment groups were formed: CS-CTIF/B (*n* = 118), CS-CT5FU (*n* = 78), PS-CTIF/B (*n* = 77), and PS-CT5FU (*n* = 64). The tumors were staged according to the World Health Organization (WHO). Compared with surgery alone, both the CS-CTIF/B and CS-CT5FU treatment groups showed higher rates of local disease-free and metastasis-free status. Furthermore, all adjuvant treatment groups demonstrated a significant improvement in overall survival compared with their respective control groups. The study was limited by the lack of immunological assessments, including immunophenotyping, cytokine profiling, and evaluation of immune correlates of protection, as well as the absence of evaluation of potential systemic immune responses against xenoantigens following repeated vaccination [6].

##### Immune Checkpoint Inhibitors

A variety of immune checkpoint inhibitors, including canine chimeric anti-programmed death ligand 1 (PD-L1) monoclonal antibodies (mAbs) and anti-programmed cell death protein 1 (PD-1) mAbs, are currently being evaluated in clinical trials in veterinary oncology. In a prospective study by Igase et al., treatment with the PD-1 inhibitor 4F12-E6 resulted in an MST of 166 days in 17 dogs with predominantly stage IV disease, compared with an MST of 55 days in 23 control dogs [64].

Although tumor-infiltrating lymphocytes are established predictive biomarkers of immune checkpoint inhibitor therapy in human oncology, veterinary evidence remains limited, and no corresponding predictive biomarkers have yet been validated in dogs.

### 5.3. Novel Therapies

The mutational landscape of oral melanoma shares features with UV-independent human cutaneous, mucosal, and uveal melanoma. Both canine oral melanoma and human melanoma also show activation of the MAPK and PI3K/AKT/mTOR signaling pathways [100].

Kim et al. investigated the antitumor activity of abemaciclib in vitro and in a xenograft model using 5 canine melanoma cell lines (CMeC1, KMeC, LMeC, UCDK9M4, and UCDK9M5). Treatment with abemaciclib induced G1 cell cycle arrest, modulated the expression of cell cycle-related genes and proteins, and activated autophagy without inducing apoptosis. Furthermore, abemaciclib significantly suppressed tumor growth in vivo [101].

Cyclooxygenase-2 expression has been reported to correlate with tumor cell proliferation and survival in canine melanoma tissues and cell lines. However, clinical trials have not yet evaluated the efficacy of nonsteroidal anti-inflammatory drugs in dogs with melanoma [26].

### 5.4. Treatment of Feline Melanoma

Ocular melanocytic tumors, particularly iris tumors, are the most frequently observed in cats [27]. Enucleation is the standard treatment for feline diffuse iris melanoma; data supporting surgical iridectomy or photocoagulation are lacking. These globe-preserving approaches are generally not recommended for diffuse iris melanoma because of the disease’s high metastatic potential and diffuse nature [102].

Feline oral melanoma appears more radioresistant than canine oral melanoma. Farrelly et al. [89] retrospectively evaluated five cats with oral melanoma treated with a hypofractionated RT protocol (cumulative dose, 24 Gy in three 8-Gy fractions on days 0, 7, and 21). Clinical responses were less favorable than canine outcomes reported with the same protocol, with an overall response rate of 60% (3/5), a complete response rate of 20% (1/5), and a partial response rate of 40% (2/5). The MST was 146 days. The protocol was well tolerated, with no significant acute or chronic adverse effects. Although these findings suggest that higher radiation doses or modified fractionation schemes may warrant further investigation, the very limited sample size precludes definitive conclusions regarding feline melanoma radiosensitivity or optimal protocol [89].

Little et al. described a 4-year-old Domestic Shorthair cat with a 7-mm melanoma of the nasal planum that was treated with two sessions of intravenous bleomycin-based ECT [103]. Following the second treatment, the tumor was no longer visible. At the 292-day follow-up examination, no local recurrence was detected, and the cat remained clinically healthy [103]. This case suggests the potential applicability of ECT for feline nasal melanoma. However, because the patient was relatively young, exhibited no clinical signs attributable to the mass, and had no concurrent diseases or other relevant medical history, the findings should be interpreted with caution. Further studies involving larger cohorts of cats are needed to confirm the efficacy of ECT in feline melanoma.

No established adjuvant therapies are available for metastatic feline melanoma. The Oncept vaccine appears safe in cats, with minimal reported adverse effects, but data on its effect on survival in cats with melanoma are lacking [26].

## 6. Prognosis

### 6.1. Tumor Location

Tumor location is one of the most important prognostic factors in canine melanoma. Oral and mucosal melanomas are associated with the poorest prognosis because of aggressive local invasion and high metastatic potential. Reported MST ranges from 3 to 24 months according to clinical stage. Although oral melanocytomas have occasionally been reported, their biological behavior remains unpredictable [9].

Melanomas of the lips or tongue generally have a more favorable prognosis than melanomas at other intraoral sites. In contrast, haired cutaneous melanomas are often cured by complete surgical excision with histologically tumor-free margins. Digital melanomas are malignant in approximately 86% of cases, whereas subungual melanomas are considered malignant in nearly all cases (100%) [9].

### 6.2. Traditional World Health Organization (WHO) Staging System

The traditional World Health Organization (WHO) staging system is widely used to assess prognosis in canine melanoma. It is based on three major factors: primary tumor size, regional lymph node involvement, and the presence of distant metastasis. Higher stage is consistently associated with shorter survival and a greater risk of metastatic disease [9].

The traditional WHO TNM-based staging system provides a practical framework for clinical staging of canine oral melanoma. However, this system does not incorporate several clinicopathologic factors that may provide additional prognostic information. Histologic features such as mitotic activity have been investigated as potential prognostic factors, although the strength and consistency of the available evidence vary among studies. Recent systematic evaluations have therefore suggested that these factors may complement rather than replace traditional TNM staging, and further validation is needed before they can be incorporated into a standardized clinical staging system.

### 6.3. Histologic Grading and Prognostic Markers

Histologic grading is particularly important for predicting the biological behavior of well-differentiated melanocytic tumors. Histopathologic prognostic indicators in canine melanoma include nuclear atypia, mitotic count, degree of pigmentation, depth or extent of tissue infiltration, vascular invasion, and ulceration [9].

#### 6.3.1. Mitotic Count and Mitotic Index

Mitotic count is the number of mitotic figures counted in 10 high-power fields (HPFs) at ×400 magnification using a ×40 objective, with counting performed in the area of highest mitotic activity. In contrast, mitotic index is the number of mitotic figures relative to the total number of cells within a defined tumor area or volume, although definitions vary among studies. Cutaneous tumors with a mitotic count of ≥3 per 10 HPFs (2.37 mm^2^) are generally associated with reduced 2-year survival (*n* = 226). A mitotic count of ≥4 per 10 HPFs (2.37 mm^2^) has been identified as the optimal prognostic threshold for oral and lip melanocytic tumors, predicting an increased risk of death within 1 year after diagnosis with 90% sensitivity and 84% specificity (*n* = 80) [39].

#### 6.3.2. Nuclear Atypia

Nuclear atypia is most accurately quantified by counting atypical nuclei among 200 cells and calculating the percentage of atypical nuclei. Using the criteria of Spangler and Kass, threshold values of 20% atypical nuclei for cutaneous melanocytic tumors showed 80% sensitivity, 94.4% specificity, and an overall correct classification rate of 93.3% for malignant behavior (*n* = 226), whereas a threshold of 30% atypical nuclei classified 86.3% of tumors with respect to 1-year outcome for canine oral or lip melanocytic neoplasms (*n* = 80) [41]. In a study of oral melanoma, nuclear atypia was classified into three groups using a semiquantitative decile scale: scores of 0 to 3, 4 to 8, and 9 to 10 were considered mild, moderate, and severe, respectively. Moderate to severe nuclear atypia was significantly associated with an increased likelihood of death within 1 year after diagnosis compared with mild atypia [104].

#### 6.3.3. Degree of Pigmentation

Pigmentation has been reported to have a significant inverse correlation with survival, but its prognostic value is limited except in tumors with very high pigment values, which are associated with longer survival [104]. Although these studies support an association between greater pigmentation and longer survival, this parameter cannot be assessed reliably because there are no objective measurement methods or validated cutoff points for comparative evaluation [39].

#### 6.3.4. Level of Infiltration/Invasion

The prognostic significance of bone lysis in canine oral melanoma remains controversial. Although some studies found no association between radiographic bone lysis and clinical outcome, another study demonstrated that the absence of bone lysis was significantly associated with a longer time to first event and survival [9,105,106]. Thus, bone lysis is not considered a consistent prognostic indicator because of conflicting findings among studies. These discrepancies may be related to differences in tumor populations, treatment approaches, and disease burden.

In contrast, the depth of local invasion has shown greater prognostic relevance in canine cutaneous melanocytic neoplasms. In a retrospective study of 68 canine cutaneous melanocytic tumors, lesions confined to the dermis have been associated with longer survival (*p* < 0.0001), whereas deeper invasion has been identified as an independent negative prognostic factor (*p* = 0.0012) [107]. Earlier studies evaluated the anatomical depth of invasion, whereas more recent studies have assessed tumor thickness as a quantitative prognostic parameter. In a retrospective study of 77 canine primary cutaneous melanocytic tumors, greater tumor thickness was associated with shorter overall survival and disease-free time. Cutoffs of 0.95 cm and 0.75 cm were identified for unfavorable clinical outcome (tumor-related death) and recurrence/metastasis, respectively [74]. These study-specific cutoffs require further validation in larger cohorts.

Vascular invasion involving either blood or lymphatic vessels is considered a strong histologic indicator of malignant behavior in both canine cutaneous and oral/lip melanocytic neoplasms. Its presence supports a diagnosis of malignant melanoma even when other histologic parameters do not meet the established criteria for malignancy [36].

#### 6.3.5. Margin Evaluation

In canine oral malignant melanoma, one study reported that curative-intent excision, which aimed to include at least 1.5–2 cm of macroscopically normal bone, soft tissue, or both around the tumor depending on its location, was associated with a significantly longer disease-free interval than marginal excision (232 vs. 183 days, *p* = 0.008) [65]. In contrast, a separate study found no significant association between complete surgical margins and either survival time or progression-free survival in dogs with oral malignant melanoma [108]. These discrepancies may be related to differences in the definition and assessment of surgical margins, treatment approaches, and patient populations.

### 6.4. Molecular Prognostic Parameters

Several immunohistochemical markers have also been investigated as prognostic biomarkers. Ki-67, particularly when evaluated in primary tumors, has demonstrated prognostic value in both oral and cutaneous melanoma. Other potential prognostic biomarkers include cyclooxygenase-2, KIT, matrix metalloproteinases, epidermal growth factor receptor, and SOX family proteins, although their clinical utility requires further validation [36].

Cell cycle phase index measured by bromodeoxyuridine or proliferating cell nuclear antigen (PCNA) labeling may have some prognostic significance in melanoma, but the available studies are too limited to support specific conclusions or recommendations [9]. In a retrospective study, Roels et al. reported significantly higher PCNA immunoreactivity in melanoma than in melanocytoma. However, PCNA expression intensity did not have significant prognostic value [109].

#### 6.4.1. Growth Fraction

The Ki-67 index reflects the growth fraction of a tumor by detecting cells in all phases of the cell cycle except the resting phase. Ki-67 expression has been reported to differ significantly between melanocytoma and melanoma [109]. In cutaneous melanomas, the Ki-67 index is calculated as the percentage of Ki-67-positive cells among 500 tumor cells, whereas in oral and lip melanocytic neoplasms, it is determined as the mean number of Ki-67-positive cells within a 1 mm^2^ optical grid reticle at ×400 magnification [104,107]. Although further validation is warranted, the Ki-67 index offers the advantage of being less observer-dependent and more objective than the subjective evaluation of qualitative histologic features, although further efforts are needed to establish standardized evaluation methods [32].

#### 6.4.2. Platelet-Derived Growth Factor Receptor (PDGFR)

Platelet-derived growth factor receptor (PDGFR) has been proposed as an emerging prognostic marker involved in the pathogenesis of melanoma. Its expression in both tumor cells and the stromal compartment suggests a potential role in extracellular matrix remodeling and tumor invasion [110]. Iussich et al. proposed that the co-expression of PDGFR-α and PDGFR-β may be associated with an unfavorable prognosis and could therefore be considered a potential negative prognostic marker [110,111].

## 7. Conclusions

Canine and feline melanocytic tumors show marked species- and site-dependent differences in prevalence and biological behavior, with canine oral and digital melanomas generally exhibiting aggressive metastatic potential, haired-skin tumors often showing more indolent behavior, and feline melanomas occurring less commonly and predominantly involving ocular sites.

An accurate diagnosis of melanoma is essential for selecting appropriate treatment strategies and providing reliable prognostic assessment. Although histopathology remains the gold standard for diagnosis, continued refinement and validation of ancillary diagnostic tools, including immunocytochemistry and immunohistochemistry, are important, particularly for poorly differentiated or amelanotic tumors. More recently, advances in molecular diagnostics, including cfDNA analysis and genomic profiling, have opened new opportunities for identifying biomarkers that may extend beyond diagnosis to facilitate disease monitoring, prognostic assessment, and personalized therapeutic decision-making.

Surgery remains the cornerstone of treatment for canine and feline melanoma, while radiation therapy and other local modalities provide additional options for unresectable or incompletely excised tumors. However, the survival benefit of currently available systemic therapies remains uncertain.

Although both canine and feline melanomas are addressed in this review, the available evidence is considerably more extensive in dogs. Further feline-specific studies are needed to expand the current evidence base and better define treatment outcomes.

## Figures and Tables

**Table 1:** Biological behavior of canine melanocytic tumors according to anatomical site.

Anatomical Site	Biological Behavior
**Oral cavity**	Generally reported as highly aggressive, with most lesions being malignant and showing a high metastatic potential [9,17,19]
**Lip**	Approximately 70% of lesions have been reported as malignant, representing a lower proportion of malignant lesions than that reported for lesions in the oral cavity [20]
**Haired skin**	Usually indolent, with most lesions reported to be biologically benign [9,21]
**Digit**	Often aggressive, with metastasis reported in approximately 50% of cases [22]
**Scrotum**	Approximately 40% of lesions have been reported as malignant; malignant lesions may show more rapid growth and ulceration [23]

**Table 2:** Traditional World Health Organization TNM-based staging scheme for dogs with oral melanoma.

**T:**	**Primary tumor**	
	**T1**	**Tumor < 2 cm**
	**T2**	**Tumor 2–4 cm**
	**T3**	**Tumor > 4 cm**
**N:**	**Regional lymph nodes**	
	N0	No evidence of regional node involvement
	N1	Histologic/cytologic evidence of regional node involvement
	N2	Fixed nodes
**M:**	Distant metastasis	
	M0	No evidence of distant metastasis
	M1	Evidence of distant metastasis

Stage I = T1 N0 M0; Stage II = T2 N0 M0; Stage III = T2 N1 M0 or T3 N0 M0; Stage IV = Any T, any N, and M1.

## Data Availability

No new data were created or analyzed in this study. Data sharing is not applicable to this article.

## Abbreviations

The following abbreviations are used in this manuscript:

cfDNA	cell-free DNA
CR	complete response
DII	DNA integrity index
ECT	electrochemotherapy
GET	gene electrotransfer
MC	metronomic chemotherapy
MST	median survival time
OR	objective response
ORR	overall response rate
PR	partial response
PDGFR	platelet-derived growth factor receptor
